# Midgut barriers prevent the replication and dissemination of the yellow fever vaccine in *Aedes aegypti*

**DOI:** 10.1371/journal.pntd.0007299

**Published:** 2019-08-14

**Authors:** Lucie Danet, Guillaume Beauclair, Michèle Berthet, Gonzalo Moratorio, Ségolène Gracias, Frédéric Tangy, Valérie Choumet, Nolwenn Jouvenet

**Affiliations:** 1 Viral Genomics and Vaccination Unit, Institut Pasteur, UMR3569 CNRS, Paris, France; 2 Université Paris Diderot, Sorbonne Paris Cité, Paris, France; 3 Environment and Infectious Risks Unit, Institut Pasteur, Paris, France; 4 Viral Populations and Pathogenesis Unit, Institut Pasteur, UMR3569 CNRS, Paris, France; 5 Laboratorio de Inmunovirología, Institut Pasteur de Montevideo, Montevideo, Uruguay; 6 Laboratorio de Virología Molecular, Centro de Investigaciones Nucleares, Facultad de Ciencias, Universidad de la República, Montevideo, Uruguay; University of Texas Medical Branch, UNITED STATES

## Abstract

**Background:**

To be transmitted to vertebrate hosts *via* the saliva of their vectors, arthropod-borne viruses have to cross several barriers in the mosquito body, including the midgut infection and escape barriers. Yellow fever virus (YFV) belongs to the genus *Flavivirus*, which includes human viruses transmitted by *Aedes* mosquitoes, such as dengue and Zika viruses. The live-attenuated YFV-17D vaccine has been used safely and efficiently on a large scale since the end of World War II. Early studies have shown, using viral titration from salivary glands of infected mosquitoes, that YFV-17D can infect *Aedes aegypti* midgut, but does not disseminate to other tissues.

**Methodology/Principal findings:**

Here, we re-visited this issue using a panel of techniques, such as RT-qPCR, Western blot, immunofluorescence and titration assays. We showed that YFV-17D replication was not efficient in *Aedes aegypti* midgut, as compared to the clinical isolate YFV-Dakar. Viruses that replicated in the midgut failed to disseminate to secondary organs. When injected into the thorax of mosquitoes, viruses succeeded in replicating into midgut-associated tissues, suggesting that, during natural infection, the block for YFV-17D replication occurs at the basal membrane of the midgut.

**Conclusions/Significance:**

The two barriers associated with *Ae*. *aegypti* midgut prevent YFV-17D replication. Our study contributes to our basic understanding of vector–pathogen interactions and may also aid in the development of non-transmissible live virus vaccines.

## Author summary

Most flaviviruses, including yellow fever virus (YFV), are transmitted between hosts by mosquito bites. The yellow fever vaccine (YFV-17D) is one of the safest and most effective live virus vaccine ever developed. It is also used as a platform for engineering vaccines against other health-threatening flaviviruses, such as Japanese encephalitis, West Nile, dengue and Zika viruses. We studied here the replication and dissemination of YFV-17D in mosquitoes. Our data showing that YFV-17D is unable to disseminate to secondary organs, as compared to a YFV clinical isolate, agree with previous studies. We have expanded on this knowledge by quantifying viral RNA production, viral protein expression, viral distribution and infectivity of YFV-17D in the vector midguts. We show that the midgut is a powerful barrier that inhibits YFV-17D dissemination in mosquitoes. Our study contributes to our basic understanding of the interactions between viruses and their vectors, which is key for conceiving new approaches in inhibiting virus transmission and designing non-transmissible live virus vaccines.

## Introduction

Arboviruses, which are transmitted among vertebrate hosts by blood-feeding arthropod vectors, put billions of people at risk worldwide. Viral infection in arthropods is usually persistent. Following uptake of an infectious blood meal by a female mosquito, arbovirus must initiate a productive infection of the midgut epithelium, which consists of a single layer of cells [1]. To develop a disseminated infection, virus must then escape the midgut into the haemocoel and infect secondary tissues such as the fat body, trachea and the salivary glands [1]. Finaly, the virus needs to be released into salivary ducts for horizontal transmission to an uninfected vertebrate host [1]. Traditional means of controlling the spread of arbovirus infection include mosquito control and vaccination of susceptible vertebrates. However, in many cases, these measures are either unavailable or ineffective. To successfully implement the strategy of blocking the virus at the arthropod stage, further knowledge of the virus/vector interactions is required.

Flaviviruses constitute the most important and diverse group of arthropod-transmitted viruses causing diseases in humans. They are 50 nm-diameter enveloped viruses harboring a single positive-strand RNA genome of around 11 kb. The genome encodes a polyprotein that is cleaved into seven non-structural (NS) proteins (NS1, NS2A, NS2B, NS3, NS4A, NS4B, and NS5) and three structural proteins: capsid (C), pre-membrane/membrane (prM/M) and envelope (Env). The C, M, and Env proteins are incorporated into virions, while NS proteins are not [2,3]. NS proteins coordinate RNA replication, viral assembly and modulate innate immune responses.

Several members of the *Flavivirus* genus, such as dengue virus (DENV), yellow fever virus (YFV) and Zika virus (ZIKV) are highly pathogenic to humans and constitute major global health problems. YFV is responsible for viral hemorrhagic fever resulting in up to 50% fatality [4]. Despite the existence of the safe and effective live-attenuated vaccine YFV-17D, YFV regularly resurges in the African and South American continents, as illustrated by recent outbreaks in Brazil and equatorial Africa [5–7]. The YFV-17D vaccine has been used safely and efficiently on a large scale since the end of World War II [8]. It was developed in the 1930’s by passaging the blood of a human patient in rhesus macaques and later in mouse and chicken embryo tissues [9]. A single dose confers protective immunity for up to 35 years. During the attenuation process, the virus has lost its neurotropic and viscerotropic properties, which account for the major disease manifestations of yellow fever in primates [10,11]. The molecular determinants responsible for its virulence attenuation and immunogenicity are poorly understood. We have recently shown that YFV-17D binds and enters mammalian cells more efficiently than a non-attenuated strain, resulting in a higher uptake of viral RNA into the cytoplasm and consequently a greater cytokine-mediated antiviral response [12]. This differential entry process may contribute to attenuation in humans.

YFV-17D is also used as a platform for engineering vaccines against other health-threatening flaviviruses, such as vaccines against Japanese encephalitis virus (JEV), West Nile virus (WNV), the four serotypes of DENV, and, more recently, ZIKV [13–16]. These vaccines consist in a YFV-17D backbone in which sequences coding for prM/E proteins are replaced by those of the selected flavivirus. Some of these live-attenuated chimeric vaccines are commercially available [17,18], with variable success [19]. YFV-17D is thus a key component in controlling flaviviral disease and it must not disseminate in mosquitoes. Early studies have shown, using almost exclusively viral titration by plaque assays, that YFV-17D can infect *Aedes aegypti* midgut [20,21], but does not disseminate to other tissues and fails to be transmitted to a novel host. Here, we re-visited this question using a variety of techniques and showed that not only the midgut escape barrier, but also the midgut infection barrier, restrict YFV-17D replication in its vector.

## Materials and methods

### Viruses

The YFV-17D vaccine strain (YF-17D-204 STAMARIL, Sanofi Pasteur, Lyon) was provided by the Institut Pasteur Medical Center. The YFV-DAK strain (YFV-Dakar HD 1279) was provided by the World Reference Center for Emerging Viruses and Arboviruses (WRCEVA), through the University of Texas Medical Branch at Galveston, USA. Viral stocks were prepared on Vero cells, concentrated by polyethylene glycol 6000 (Sigma) precipitation and titrated on Vero cells by plaque assay as described previously [22].

### Cells

The Aag2 mosquito cell lines (provided by the teams of M. Flamand and L. Lambrechts, Institut Pasteur, Paris) are derived from larvae of *Aedes aegypti*. They were cultured in a humid chamber at 28°C, with no CO_2_, in Leibovitz medium (Gibco Leibovitz's L-15 Medium, Life Technologies) supplemented with 10% fetal bovine serum (FBS), 2% tryptose phosphate buffer (Gibco Tryptose Phosphate Broth 1X, Life Technologies), 1/100 dilution of the penicillin-streptomycin (P/S) stock (final concentration of 100 units/mL and 100 μg/mL, respectively) (Sigma) and non-essential amino acids (GibcoTM NEAA 100X MEM, Life Technologies). Vero cells, which are African green monkey kidney epithelial cells, were purchased from the American Type Culture Collection (ATCC) and used to perform viral titration. They were maintained in Dulbecco's modified Eagle's medium (DMEM, Invitrogen), supplemented with 10% FBS and 1% P/S.

### Antibodies

Env MAb 4G2 hybridoma cells were kindly provided from P. Desprès (La Réunion University, Sainte Clotilde). Anti-YFV-NS4B and anti-DENV NS1 17A12 (that recognize YFV-NS1) antibodies, were kind gifts from C.M. Rice (Rockefeller University, NY) [23] and M. Flamand (Institut Pasteur, Paris) [24], respectively. Anti-actin (A1978, Sigma) and anti-tubulin (T5168, Sigma) antibodies were used as loading controls for mosquito organs and Aag2 cells, respectively. Secondary antibodies were as followed: anti-mouse 680 (LI-COR Bioscience), anti-rabbit 800 (Thermo Fisher Scientific) and anti-rabbit Cy3 (Life Technologies).

### Infection and dissection of mosquito

The Paea strain of *Ae*. *aegypti* is a laboratory colony originated from mosquitoes collected in French Polynesia in 1960 and conserved in the laboratory since 400–450 generations. Adult mosquitoes were maintained at 25 ± 1°C and 80% relative humidity with a light/dark ratio of 12 h/12 h. The larvae were provided with brewer’s yeast tablets and adults were given continuous access to 10% sucrose solution. Sucrose was removed 24 h prior to the infectious blood meal. The infectious blood meal was comprised of half-human blood and half-viral suspension (4.10^7^ PFU/mL in the mix). The blood donors were randomly selected from a population of healthy volunteers donating blood at the ‘Etablissement Français du Sang’ (EFS), within the framework of an agreement with Institut Pasteur. Experimental procedures with human blood have been approved by EFS Ethical Committees for human research. All samples were collected in accordance with EU standards and national laws. Informed consent was obtained from all donors. Seven day-old female mosquitoes were allowed to feed for 15 min through a collagen membrane covering electric feeders maintained at 37°C (Hemotek system). Blood-fed females were selected and transferred into cardboard boxes protected with mosquito nets. Alternatively, ice-chilled mosquitoes were injected intrathoracically with twice 69 nL of viral stock (2.5x10^4^ PFU) with a micro-injector (Drummond, Nanoject II). Mosquitoes were anesthetized on ice at various time-points after infection. They were passed through a 70% ethanol bath and then in a PBS bath before being dissected in a drop of PBS under a magnifying glass using tweezers. The midguts, legs and salivary glands were removed and placed in individual tubes containing sterilized glass beads of a diameter of 0.5 mm (Dutscher) in a suitable lysis buffer. Experiments were reproduced in triplicate with 5–10 mosquitoes collected at each time-point for dissection.

### RT-qPCR analyses

The mosquito midguts, legs or salivary glands were crushed using a tissue homogenizer (Ozyme, Precellys Evolution) during twice 15 s at 1000 g. Total RNA was extracted from mosquito tissues with the NucleoSpin RNA II kit (Macherey-Nagel). YFV RNA was quantified using NS3-specific primers and TaqMan probe (NS3-For CACGGCATGGTTCCTTCCA; NS3-MFAM CAGAGCTGCAAATGTC; NS3-Rev ACTCTTTCCAGCCTTACGCAAA) with TaqMan RNA-to-CT 1-Step (Thermo Fisher Scientific‎) on a QuantStudio 6 Flex machine (Applied Biosystems). Genome equivalent (GE) concentrations were determined by extrapolation from a standard curve generated from serial dilutions of total YFV RNA of a known concentration.

### Western blot analyses

Individual midguts and salivary glands were collected in RIPA buffer (Sigma) containing protease inhibitors (Roche Applied Science). Tissue lysates were normalized for protein content with Pierce 660nm Protein Assay (Thermo Scientific), boiled in NuPAGE LDS sample buffer (Thermo Fisher Scientific) in non-reducing conditions and 32 μg (midgut) or 14 μg (salivary glands) of proteins (corresponding to around 10 pooled organs) were separated by SDS-PAGE (NuPAGE 4–12% Bis-Tris Gel, Life Technologies). Separated proteins were transferred to a nitrocellulose membrane (Bio-Rad). After blocking with PBS-Tween-20 0.1% (PBST) containing 5% milk for 1 h at RT, the membrane was incubated overnight at 4°C with primary antibodies diluted in blocking buffer. Finally, the membranes were incubated for 1 h at RT with secondary antibodies diluted in blocking buffer, washed, and scanned using an Odyssey CLx infrared imaging system (LI-COR Bioscience).

### Immunofluorescence

After dissection, individual midgut were deposited on slides, fixed in cold acetone for 15 min and rehydrated in PBS for 15 min. The midguts were then incubated for 2 h in Triton X-100 (0.2%). After washing with PBS, they were incubated for 30 min with PBS + 0.1% Tween 20 + 1% BSA. The slides were then incubated overnight at 4°C with anti-YFV-NS4B antibodies diluted 1:1000 in PBS. After washing with PBS, they were incubated for 1 h with secondary antibodies and washed with PBS. The actin network was visualized with phalloidin Alexafluor 488 (Invitrogen). After washing, nuclei were stained using Prolong gold antifade containing 4′,6-diamidino-2-phenylindole (DAPI) (Invitrogen). All preparations were observed with a confocal microscope (ZEISS LSM 700 inverted) and images were acquired with the ZEN software.

### Deep-sequencing of viral stocks

Viral RNA was extracted from viral stocks (around 1.4x10^9^ genomes for YFV-17D and 3.4x10^8^ for YFV-DAK) using Trizol (Ambion, TRIzol Reagent), were re-suspended in RNAse-free water and treated with DNAse with the DNA-free kit (Ambion) before being stored at -80°C. Synthesis of cDNAs was carried out with the Maxima H Minus First Strand kit (Thermo Fisher Scientific) from 250 ng of viral RNA. Three fragments of the viral genome were amplified by 25 rounds of PCR using the Phusion High-Fidelity DNA Polymerase kit (NEB) using primers mainly described previously [25]. New primers targeting the 3'-UTR of the genome were designed for optimal amplification of YFV-17D and YFV-DAK (Table 1). The PCR products were purified with the NucleoSpin Gel kit and PCR Clean Up (Macherey-Nagel), resuspended in RNAse-free water and stored at -20°C. Libraries were prepared after pooling 400 ng of the three overlapping amplicons, which had a size between 3725 and 3891 pb. The PCR products were fragmented randomly with the NEBNext dsDNA fragmentase kit (NEB) and then purified with the AMPure XP Beads kit (Beckman Coulter, Inc.). The Illumina sequencing libraries were prepared with the NEBNext Ultra DNA Library Prep kit (NEB) by selecting 400 bp fragments. NEBNext Multiplex Oligos for Illumina primers (NEB) were used. Purification was performed with the AMPure XP Beads kit. The Qubit dsDNA BR Assay kit (Thermo Fisher Scientific) was used for quantification. Samples from the library, diluted to 4 nM, were sequenced on a NextSeq 500 sequencer (Illumina) machine with the NextSeq 500 Mid Output Kit v2 kit (150 cycles) (Illumina), to generate single-end reads of 150 nt. The PhiX control library served as a quality and calibration control in sequencing runs (Illumina, FC-110-3001).

**Table 1:** Primers used for YFV sequencing.

Fragment length	Forward	Reverse
a *3725 pb*	AGTAAATCCTGTGTGCTAATTGAGGTG	TTGAAAAGGCAGCAATCAACGC
b *3781 pb*	GGGTTACAGCTGGAGAAATACATGC	TGCTGCGCTTTCATTCCAGGTA
c (1) *3874 pb*	TGCTGGAGAAAACCAAAGAGGA	GGTCTTTCCCTGGCGTCAATA
c (2) *3891 pb*	TGCTGGAGAAAACCAAAGAGGA	AAGCAGAGAACCACTCCGGT

Viral genome was amplified in three fragments (a, b and c) of approximately 3000 pb. Different reverse primers were used to amplify the c fragment of the two YFV strains resulting in c(1) for YFV-17D and c(2) for YFV-DAK. All primers used to amplify fragments a and b were previously described [25].

### Viral sequence analyses and comparison of diversity

Reads were trimmed for adapters and primer sequences. Low quality reads were filtered using Trim Galore! (www.bioinformatics.babraham.ac.uk/projects/trim_galore/) with the following parameters: quality 30, length 75 and stringency 4. Final reads quality was evaluated using FastQC (www.bioinformatics.babraham.ac.uk/projects/fastqc/). Reads were aligned on the YFV-Asibi reference genome AY640589.1 using BWA [26] and SAMtools [27]. Consensus sequences were obtained using SAMtools mpileup, VarScan mpileup2cns (min-var-freq 0.5) and BCFtools consensus [27]. When mapping YFV-DAK sequencing reads on the YFV-Asibi sequence, an uncomplete coverage was observed. We reiterated the mapping of YFV-DAK reads on this intermediate consensus sequence to obtain the final YFV-DAK consensus sequence. Consensus sequence of YFV-17D and YFV-DAK have been deposited on NCBI (ID numbers MN10624 and MN106242), and sequencing data have been uploaded on SRA (SRA accession number PRJNA548475). Variant determination was estimated using VarScan mpileup2snp (min-var-freq 0.01, strand-filter 0) with a cutoff of 3%.

### Statistical analyses

Data were analyzed using GraphPad Prism 7. Statistical analyses were performed using two-tailed Fisher's exact test or Mann-Whitney test (* p < 0.05; ** p < 0.01; *** p < 0.001; **** p < 0.0001, ns, not significant.), as indicated.

## Results

### YFV-17D, but not YFV-DAK, fails to overcome the midgut barriers of *Aedes aegypti*

**Fig 1:**
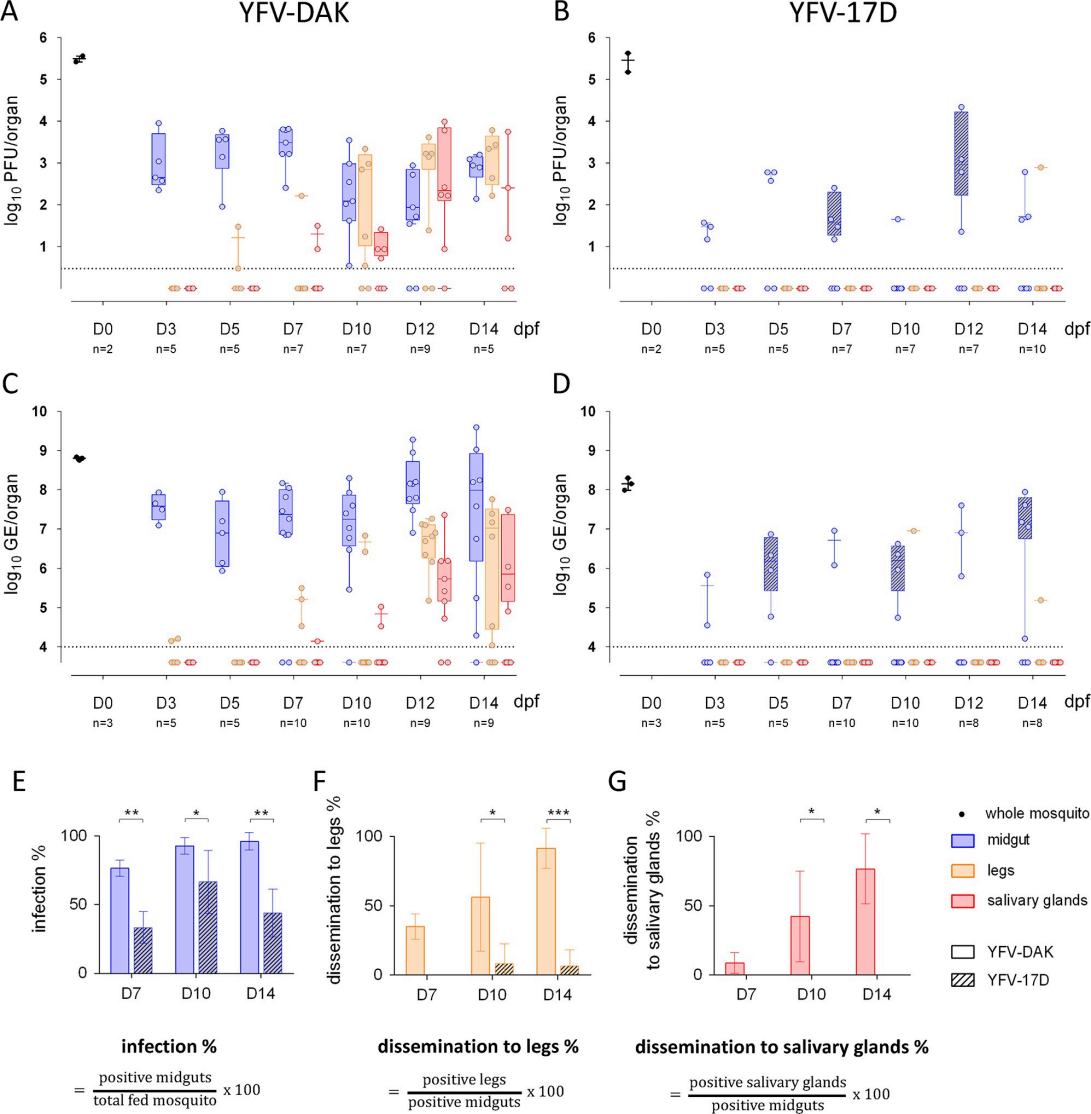
YFV-17D, but not YFV-DAK, fails to overcome the midgut barriers of *Aedes aegypti*. Mosquitoes were orally infected with 4.10^7^ PFU/mL of YFV-DAK (A and C) or YFV-17D (B and D). (A and B) The presence of infectious viruses in individual midgut, legs and salivary glands was assessed by plaque assay on Vero cells at 3, 5, 7, 10, 12 and 14 day post feeding (dpf). Several whole mosquitoes were also analyzed 20 minutes after feeding (black dots). Each data point represents the YFV titers of a single organ. (C, D) The relative amounts of organ-associated viral RNA were determined by RT-qPCR analysis and are expressed as genome equivalents (GE) per organ at 3, 5, 7, 10, 12 and 14 dpf. Total RNA was also extracted from several whole mosquitoes the same day of the feeding (black dots). (A-D) The dashed lines indicate the limit of detection. One representative RT-qPCR experiments out of three independent ones is shown. The other two are shown in S1 Fig. YFV infection rates among midguts (E), YFV dissemination rate among legs (F) and YFV dissemination rate among salivary glands (G) were determined by RT-qPCR analysis at 7, 10 and 14 dpf. (E-G) Data were obtained from 3 independent experiments. Error bars indicate the means ± SD. Statistical analyses were performed using a two-tailed Fisher's exact test (* p < 0.05; ** p < 0.01; *** p < 0.001; **** p < 0.0001).

The replication and dissemination of YFV-17D was studied in the *Ae*. *aegypti* strain Paea. The clinical isolate YFV-Dakar HD 1279 (YFV-DAK), whose replication in rhesus macaque is well characterised [28], was used as a positive control for these experiments. Virus produced on Vero cells were mixed with human blood to prepare a meal containing 4x10^7^ PFU/mL of either YFV-17D or YFV-DAK. Five to ten mosquitoes were collected every 2–3 days until 14 days post-feeding (dpf). Mosquitoes were dissected to separate the midgut from legs and salivary glands. Virus production in these tissues was first assayed by calculating the viral titer by plaque assays on Vero cells. Several whole mosquitoes were also analyzed 20 minutes after feeding to ensure that the mosquitoes ingested a similar amount of viral particles of both viral strains (Fig 1A and 1B, black dots). Around 10^3^ infectious particles of YFV-DAK were detected per midguts 3 dpf (Fig 1A). Viral titers remained high in midguts until 14 dpf. YFV-DAK infectious particles were present in legs as early as 5 dpf and in salivary glands as early as 7 dpf (Fig 1A). This replication pattern is comparable to that of South American and African YFV isolates in the strain *Ae*. *aegypti* AE-GOI [29]. Midgut of mosquitoes infection with YFV-17D produced 1 to 2 log less infectious particles than YFV-DAK at 3 dpf (Fig 1B). Infectious particles were detected in a unique leg sample at 14 dpf. No virus was detected in salivary glands of mosquitoes infected with YFV-17D. Thus, by contrast to YFV-DAK, and in agreement with previous studies performed with the *Ae*. *aegypti* strains Rexville or Rexville-D (Rex-D) [21,30–32], YFV-17D disseminated poorly in the strain Paea.

Viral replication was assessed in the midguts, legs and salivary glands by measuring viral RNA quantity over-time by RT-qPCR (Figs 1C, 1D and S1). Total RNA was also extracted from several whole mosquitoes the same day of the feeding to insure that they had ingested similar amount of infectious particles from both viral strains (Fig 1C and 1D, black dots). Around 10^7^ copies of viral RNA were detected in midguts of mosquitoes infected with YFV-DAK since 3 days (Figs 1C, S1A and S1C). The viral RNA copy number per midgut remained high until 14 dpf, indicating that viral replication had already reached a plateau at an early stage of infection (Figs 1C, S1A and S1C). In agreement with titration assays (Fig 1A), YFV-DAK RNA was detected in legs and salivary glands of mosquitoes around 7 dpf. The quantity of viral RNA detected in these secondary organs increased over time to reach on average 10^7^ copies RNA in legs and 10^6^ copies in salivary glands at 14 dpf (Figs 1C, S1A and S1C). Around 5x10^5^ copies of YFV-17D RNA was detected in 2 out of 5 midguts of blood-feed mosquitoes at 3 dpf (Fig 1D). At 12 dpf, around 10^7^ copies of YFV-17D RNA was detected in 4 out of 8 mosquitoes, which is 10 time less than in YFV-DAK infected moquitoes. YFV-17D RNA was found in legs of 2 mosquitoes among the 46 blood-fed mosquitoes collected during 14 days (Figs 1D, S1B and S1D). No virus was dectected in the salivary glands of these 46 mosquitoes (Figs 1D, S1B and S1D). Therefore, in agreement with our titration assays (Fig 1B) and with previous studies performed with Rexville strains of *Ae*. *aegypti* [30–32], YFV-17D disseminated poorly in the strain Paea. The RT-qPCR analyses also revealed that the vaccine strain replicated less efficiently than YFV-DAK in its vector.

The percentage of mosquitoes that were positive for viral RNA among the mosquitoes that had taken blood was calculated based on RT-qPCR data obtained from 3 independent experiments (Fig 1E). Significantly less midguts were postivive for YFV-17D RNA than YFV-DAK RNA at days 7 and 14 post-feeding, suggesting that the midgut infection barrier restricts the replication of the vaccine strain. Viral dissemination to legs was defined by the presence of viral RNA in the legs of mosquitoes whose midguts were infected (Fig 1F). YFV-DAK had disseminated in around 40% of infected mosquitoes at 7 dpf and in around 90% of mosquitoes at 14 dpf. At this time, YFV-17D had disseminated in around 10% of them (Fig 1F). YFV-DAK dissemination rates are consistent with the ones reported for the YFV-Asibi strain [32] or clinical isolates from Peru [31] in Rexville mosquitoes. YFV-DAK RNA was detected in salivary glands of approximately 75% of mosquitoes whose midguts were infected, revealing that the virus had efficiently reach these secondary organs (Fig 1G). By constrast, no dissemination in salivary glands was observed in mosquitoes infected with YFV-17D.

**Fig 2:**
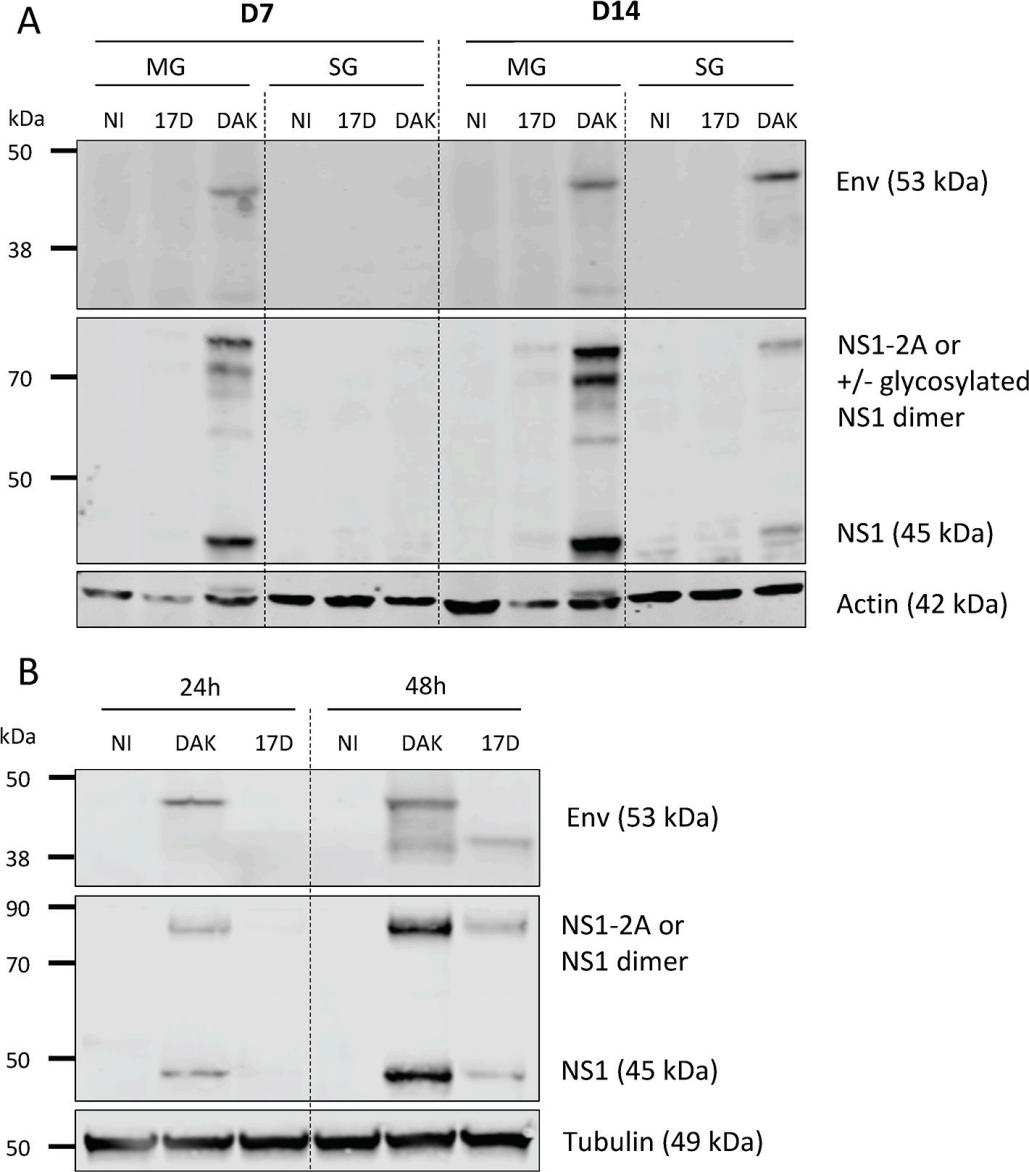
YFV-DAK produces detectable levels of viral proteins in infected mosquitoes. (A) Mosquitoes were fed with human blood containing 4.10^7^ PFU/mL of YFV-DAK or YFV-17D or no virus (NI). The presence of viral antigens in 10 pooled midguts and salivary glands was analyzed by immunoblotting at 7 and 14 dpf using antibodies recognizing actin, viral NS1 or Env proteins. (B) Aag2 cells were infected at a MOI of 1. Whole-cell lysates were analyzed by immunoblotting at the indicated times post-infection using antibodies recognizing human tubulin, viral NS1 or Env proteins. Non-reducing condition were used to detect the Env proteins.

To investigate the replication ability of the two viral strains further, the presence of viral antigens in pooled midguts and salivary glands of mosquitoes fed on blood containing 4.10^7^ PFU/mL of either YFV-17D or YFV-DAK was analyzed by Western blots at days 7 and 14 post-feeding using antibodies against Env and NS1. The Env protein was detected at both time-points in the midgut of mosquitoes infected with the strain YFV-DAK, in a majority form of around 45 kDa and a minor form of around 35 kDa (Fig 2A). The Env protein was not detected in the salivary glands 7 days after the blood meal but was present as a 45 kDa form 14 days after the blood meal (Fig 2A). These data are in good agreement with the titration and RT-qPCR data presented in Figs 1 and S1. Like the Env protein, the NS1 protein was detected in the midguts of mosquitoes infected with the YFV-DAK strain at both 7 and 14 dpf (Fig 2A). In midguts, NS1 was detected at the expected size of 45 kDa, but also as heavier forms of around 80 kDa. These forms could represent NS1-2A, a polyprotein precursor consisting of NS1 and a portion of NS2A. This NS1-2A form was previously reported in human SW-13 cells infected with YFV-17D [23] and maybe generated by alternative cleavage sites in the NS2A region upstream from the cleavage site generating the N-terminus of NS2B. Alternatively, they could represent glycosylated versions of NS1 monomer or dimer. NS1 was also detected in the salivary glands of mosquitoes infected with YFV-DAK for 14 days (Fig 2A). No or very little signal was detected by the anti-NS1 or anti-Env antibodies in organs of mosquitoes infected with YFV-17D (Fig 2A). In order to ensure that the antibodies directed against the NS1 and Env proteins recognize YFV-17D proteins, control experiments were performed with the *Ae*. *aegypti* Aag2 cells infected for 24 or 48 hours at an MOI of 0.1 with both viral strains. Both proteins were well detected in cells infected for 48 hrs, independently of the viral strain used (Fig 2B). Thus, absence of detection of YFV-17D Env and NS1 proteins in the mosquito organs at 7 and 14 dpf is not due to poor recognition of the viral antigens, nor the antibodies used, but reflects a low-level replication. These data confirm our titration and RT-qPCR analyses (Fig 1). Of note, the YFV-DAK Env was detected as 2 forms in Aag2 cells infected for 48 hours while the YFV-17D Env was detected as a unique form. No YFV-17D proteins were detected at 24 hours post-infection, suggesting that the replication of the vaccine strain is slower in Aag2 cells than the one of YFV-DAK, as in *Ae*. *aegypti* (Fig 1).

**Fig 3:**
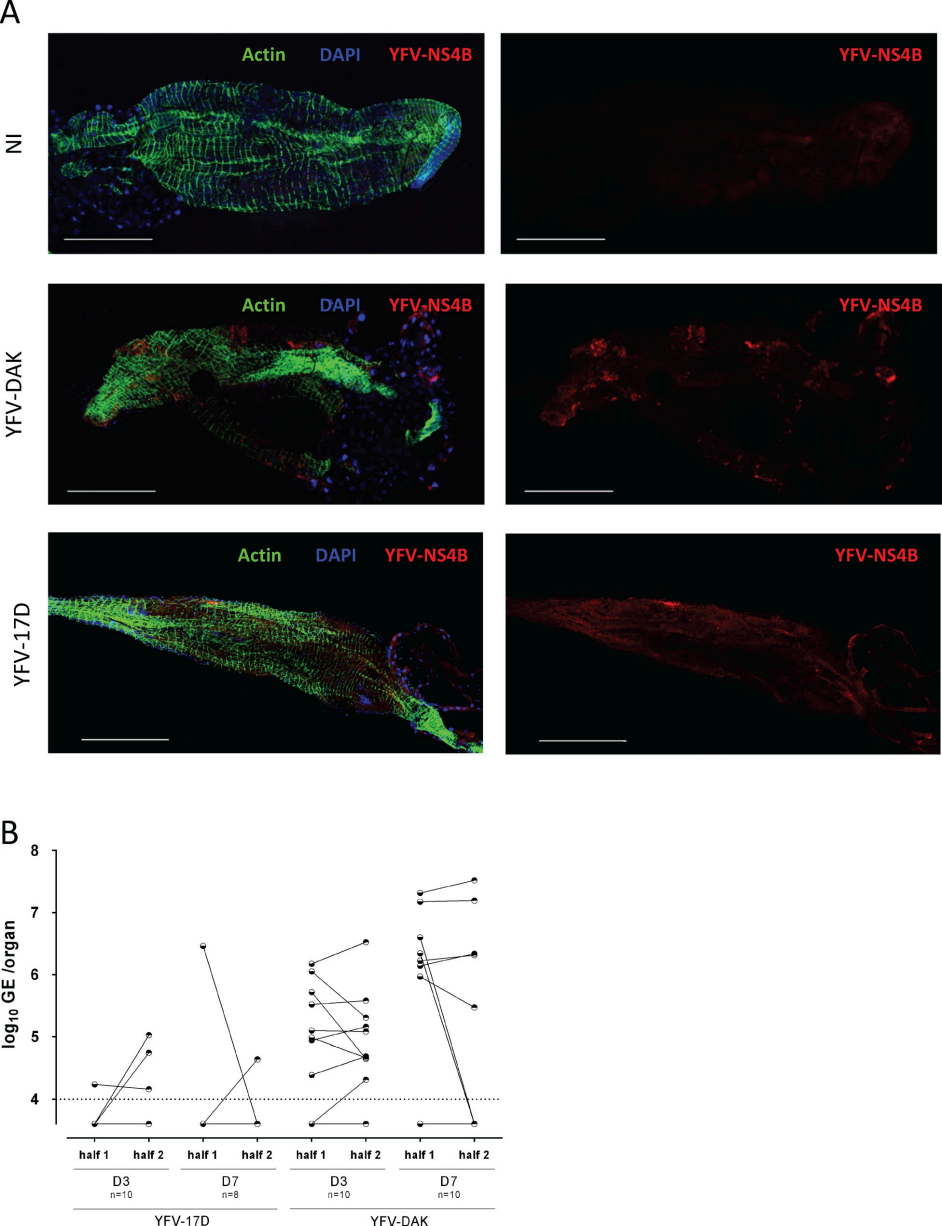
Replication of YFV-17D in midguts is localized to confined area. Mosquitoes were fed with human blood containing 4x10^7^ PFU/mL of YFV-DAK, YFV-17D or no virus (NI). (A) Midguts were dissected at 7 dpf and stained with DAPI to visualize nuclei (blue), Phalloidin Texas Red to visualize actin (green) and with antibodies recognizing the viral protein NS4B (red). Images were acquired with a confocal microscope equipped with a x40 objective. Scale bars are 0,5 mm. (B) The midguts of infected mosquitoes were cut longitudinally into two parts at 3 or 7 dpf. The presence of viral RNA was determined by RT-qPCR analysis performed on individual half midguts. The data are expressed as genome equivalents (GE) per organ. The dashed line indicates the limit of detection.

Finally, to confirm RT-qPCR and Western blot data, immunofluorescence analyses were performed on midgut of mosquitoes fed since 7 days using antibodies againt the viral protein NS4B. YFV-DAK antigens were evenly distributed in foci over the entire epithelium at this time (Fig 3A). By contrast, YFV-17D antigens were found in one or two localized foci in infected midguts. In an attempt to investigate further this uneven distribution of YFV-17D replication sites, the midgut of mosquitoes infected with both viral strains for 3 or 7 days were cut longitudinally into two equal parts. The presence of viral RNA was determined by RT-qPCR analyses performed on individual half midguts (Fig 3B). Among 18 mosquitoes that ingested blood containing YFV-17D, 3 half midguts were positive for YFV-17D RNA at day 3 post-infection and only 2 at day 7 post-infection. This is in agreement for our previous results (Fig 1E). Among these five positive midguts, only one contained YFV-17D RNA in both sections (Fig 3B). As expected based on previous results (Fig 1E), it was easier to obtain midguts positive for YFV-DAK. Twelve out of the 15 midguts that were positive for YFV-DAK RNA contained viral RNA in both sections. These experiments revealed that YFV-17D replication in *Ae*. *aegypti* midgut is more confined than YFV-DAK replication.

Together, these data show that, by contrast the clinical isolate YFV-DAK, the vaccine strain replicated poorly in, and disseminated poorly from *Ae*. *aegypti* midgut.

### YFV-17D and YFV-DAK replicate in the midgut when mosquitoes are inoculated intra-thoracically

**Fig 4:**
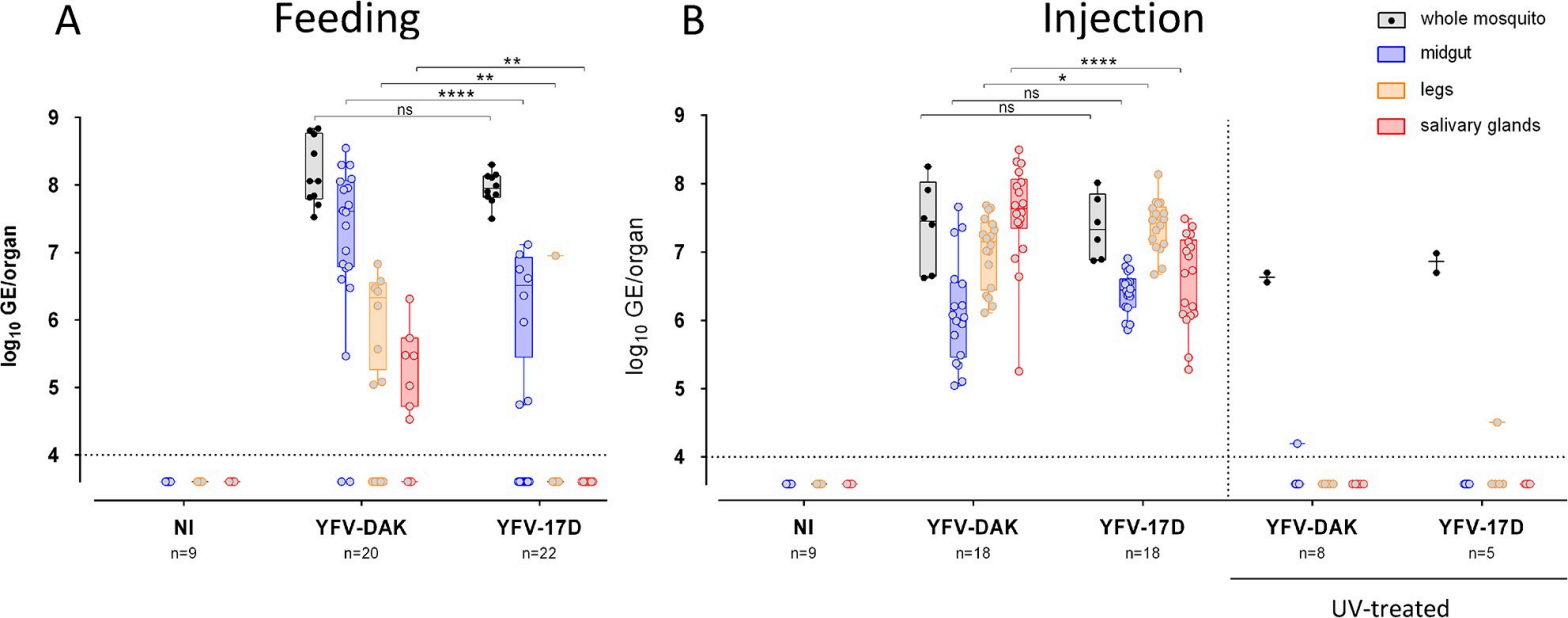
YFV-17D and YFV-DAK replicate in secondary organs when inoculated intra-thoracically. (A) Mosquitoes were orally infected *via* a blood meal containing 4.10^7^ PFU/mL of YFV-DAK, YFV-17D or no virus. Alternatively (B), mosquitoes were inoculated intra-thoracically with 2.5x10^4^ PFU of YFV-17D or YFV-DAK or with the same amount of UV-treated viruses. The relative amounts of organ-associated viral RNA were determined by RT-qPCR analysis 10 days after infection and are expressed as genome equivalents (GE) per organ. Several whole mosquitoes were also analyzed the day of the feeding or injection to ensure that a similar amount of viral particles of both viral strains were delivered in mosquitoes (black boxes). The number of organs (n) analyzed is indicated. The dashed lines indicate the limit of detection. Three independent experiments were performed with untreated viruses. Control experiments with UV-treated viruses were performed once. Statistical analyses were performed using a Mann-Whitney test (* p < 0.05; ** p < 0.01; *** p < 0.001; **** p < 0.0001).

To assess whether YFV-17D could infect *Ae*. *aegypti* when delivered *via* a non-oral route, mosquitoes were inoculated intra-thoracically with 2.5x10^4^ PFU of YFV-17D or YFV-DAK, which corresponds to around 10 times less PFU than when mosquitoes are taking around 5 μL of a blood meal containing 4x10^7^ PFU/mL. The presence of viral RNA was analyzed by RT-qPCR 10 days after injection. Mosquitoes infected *via* a blood meal served as controls. Several whole mosquitoes were also analyzed 20 minutes after feeding or injection to ensure that a similar amount of viral particles of both viral strains were delivered in mosquitoes (Fig 4, black boxes). In good agreement with our previous experiments (Fig 1E), around 35% of midguts (8 out 22) were positive for YFV-17D RNA, whereas 81% (18 out 22) were positive for YFV-DAK RNA at day 10 post feeding (Fig 4A). Moreover, significantly less viral RNA (around 10 times) was found in YFV-17D-infected midguts as compared to YFV-DAK-infected midguts (Fig 4A). YFV-DAK RNA was detected in legs and salivary glands of around 50% of these mosquitoes. By contrast, YFV-17D was detected in the legs of a unique mosquito out of 22 and was not detected in salivary glands (Fig 4A), confirming the inability of the vaccine strain to spread to secondary organs when orally delivered. When the midgut barriers were bypassed by injecting *Ae*. *aegypti* mosquitoes in the thorax, 100% of midguts were positive for both viral strains and similar amounts of YFV-17D and YFV-DAK RNA were detected in this organ, indicating that both viral strains successfully replicated in midgut-associated tissues when bypassing the lumen (Fig 4B). All legs and salivary glands were positive for YFV-17D and YFV-DAK RNA (Fig 4B), revealing that the two viruses were efficiently infecting secondary tissues once the midgut was bypassed. Of note, significantly more (around 10 times) YFV-DAK RNA was detected in salivary glands than YFV-17D RNA (Fig 4B), suggesting that YFV-17D is sensitive to the salivary gland infection barrier.

To ensure that viral RNA detected in secondary organs of injected mosquitoes represented replicative RNA and not input viral RNA, UV-treated viral RNA was also injected into the thorax of several mosquitoes. A signal, slightly above the detection threshold, was detected in two organs out of 39 tested (Fig 4B). These data confirm the ability of YFV-17D to replicate as efficiently as YFV-DAK in midgut and secondary organs when mosquitoes were inoculated intra-thoracically.

### Sequencing and single-nucleotide polymorphism frequency of YFV-17D and YFV-DAK

**Fig 5:**
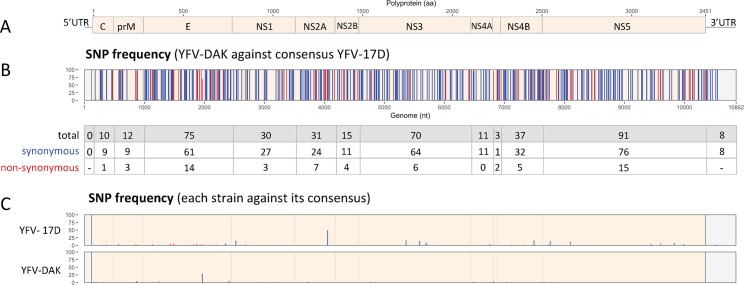
Sequencing and single-nucleotide polymorphism frequency of YFV-17D and YFV-DAK. (A) Schematic representation of YFV genome. (B) Single-nucleotide polymorphism (SNP) frequency between YFV-Dakar and the consensus sequence of YFV-17D are shown. Blue and red bars represent synonymous and non-synonymous variants, respectively. The table shows the number of SNP per coding or non-coding region. Only SNPs of more than 3% were represented. (C) Genomic intra-variability of YFV-17D and YFV-DAK. SNP frequency were obtained using the respective consensus sequence of each strain as a reference.

To determine the consensus sequence of the two viral strains, we performed next generation sequencing (NGS) analysis of the two viral stocks. Average coverage depths for these alignments were around 1000x (S1 Table) and homogeneous along their references. The comparison of the two consensus sequences identified 333 synonymous mutations (Fig 5A and 5B, blue bars) and 60 non-synonymous ones (Fig 5B, red bars). These differences were scattered along the genome. Single nucleotide variants (SNVs) and their frequency were identified all along the two genomes (Fig 5C). Only the SNVs representing a minimum of 3% of all observations were considered. The genome of YFV-17D contained more SNVs than the one of YFV-DAK (50 against 18). A SNV that lies in the NS2A gene of YFV-17D is represented in 44% of the population, but does not induce amino acid change.

## Discussion

Studies conducted shortly after the development of YFV-17D showed that *Ae*. *aegypti* fed on vaccinated volunteers or rhesus monkeys were unable to transmit YFV-17D to susceptible monkeys [33]. These results were confirmed five decades later by showing that suckling mice bitten by *Ae*. *aegypti* infected with YFV-17D did not exhibit sign of disease [31]. Poor dissemination of YFV-17D to mosquito heads was shown by examining head tissues by immunofluorescence or immunohistochemical studies [31,34]. Consistently, titration assays performed on organs of the Rex-D strain of *Ae*. *aegypti* revealed that YFV-17D infects the midgut, but does not spread to secondary organs [21,32]. Our RT-qPCR, immunofluorescence and titration analyses document the inability of YFV-17D to disseminate in the Paea strain of *Ae*. *aegypti*. Our analysis also revealed that YFV-17D replicates poorly in the midgut, as compared to the clinical isolate YFV-DAK. Among the mosquito with midgut positive for YFV-17D RNA, only 10% had viruses that disseminated to their legs and none had viral RNA in their salivary glands. Thus, our data suggest that the YFV-17D strain is not only sensitive to the midgut escape barrier, but also to the midgut infection barrier when orally delivered. When injected into the thorax of mosquitoes, YFV-17D replicated in midgut tissues as efficiently as YFV-DAK. These data suggest that the restriction of YFV-17D replication in the midgut occur at the level of epithelial cells. Our RT-qPCR analyses suggest that the major restriction occurs at a stage prior to viral RNA production. Several mechanisms, not mutually exclusive, could explain this restriction.

First, the restriction could occur during viral entry in midgut epithelial cells. The low number of loci revealed by immunofluorescence analysis of YFV-17D-infected midguts suggests that only few cells were initially infected by the vaccine strain and thus supports the hypothesis of an entry defect. *Flavivirus* entry mechanisms are poorly described in mosquito cells. Neither attachment factor(s) nor entry receptor(s) are identified yet. As in mammalian cells, the domain III of Env is involved in attachment and entry of flavivirus in mosquito cells [35]. Thus, it is conceivable that YFV-17D Env would have a lower affinity for cell entry factors than YFV-DAK Env. Our NGS analysis revealed that the consensus sequence of the two Env proteins differs from 75 mutations, including 14 non-synonymous mutations. Seven of these non-synonymous mutations lie within the domain III. Finally, in Aag2 cells infected for 48 hours, we detected two forms of YFV-DAK Env under non-reducing conditions and a single form of YFV-17D Env. These differences may reflect a different conformation and may explain a different affinity for a cell entry receptor. In agreement with this hypothesis, when domain III of the Env gene of a YFV able to disseminate was replaced by domain III of the Env gene of YFV-17D, the dissemination of the chimeric virus was strongly inhibited, suggesting an important role in Domain III in this process [21]. These results, however, may be the consequence of chimerization, as it is known that flavivirus chimeras replicate less efficiently than parental viruses [36]. We have recently shown that the YFV-Asibi enters a panel of human cells by canonical endocytosis mechanisms involving clathrin, while YFV-17D enters cells in a clathrin-independent manner [12]. We have shown that the 12 mutations differentiating YFV-Asibi Env from YFV-17D Env are responsible for the differential internalization process. Based on these data, we hypothesized that YFV-17D and YFV-Asibi use different cell receptors [12]. It is therefore possible that the YFV-17D and YFV-DAK strains also use different receptors in mosquito cells and that the receptor used by YFV-17D is poorly expressed at the apical surface of midgut epithelial cells, as compared to the one used by the clinical strain. This hypothesis is consistent with our data showing that YFV-17D succeeded in replicating into midgut-associated tissues when inoculated intra-thoracically. Alternatively, the glycosylation status of the Env protein could play a role in the differential entry abilities of the two strains. *Flavivirus* Env proteins possess a conserved N-glycosylation motif at amino acid 153/154. This modification is involved in important viral replication and pathogenesis functions [37]. Mutagenesis studies on many flaviviruses, including the DENV, WNV and ZIKV, indicate that the loss of this N^153/154^-glycosylation impairs viral replication in the midgut [38–40]. Unlike most flaviviruses, the YFV Env lacks the N^153/154^-glycosylation canonical site. A second non-canonical N-glycosylation site exists at position 470. However, it is unlikely that this site is functional because it is located in the hydrophobic carboxy-terminal domain and is therefore inserted into the endoplasmic reticulum membrane. The absence of an accessible N-glycosylation site in YFV-DAK Env therefore indicates that such motif is probably not necessary for replication and dissemination in mosquitoes. We therefore believe that mutations in Env, rather than its glycosylation status, are involved in vector competence.

Another mechanism that could explain the low replication of YFV-17D in the midgut of mosquitoes is its inability to escape the antiviral mechanisms in midgut epithelial cells. The RNA interference pathway (RNAi), is a major antiviral defense initiated by the recognition of viral replication intermediates by the Dicer-2 protein [41]. Its efficacy differs within organs, both in *Anopheles gambiae* [42] and *Aedes aegypti* [43]. One can envisage that the pathway is particularly efficient against YFV-17D in midgut epithelial cells. This pathway inhibits the replication of DENV and ZIKV viruses in the midgut and salivary glands of mosquitoes [44–46]. Interestingly, Myles and colleagues recently showed that the YFV C protein counteracts the RNA interference pathway in *Ae*. *aegypti* by protecting double-stranded viral RNA from Dicer-2-induced cleavage [47]. No amino acid sequence responsible for this effect has been identified. Our NGS analysis revealed that the consensus sequence of the C gene of our two strains of interest differ by 10 mutations, including a non-synonymous one. This unique mutation in C could modulate its RNA interference suppression activity.

The NS1 protein, which is a highly conserved glycoprotein secreted by flavivirus-infected cells, enhances DENV and JEV replication in their vectors [48]. It does so by allowing them to escape two important antiviral mechanisms: the production of reactive species of oxygen (ROS) and the JAK/STAT pathway [48]. One can envisage that, like the NS1 proteins of DENV and JEV, YFV-DAK NS1 protein could be a potent suppressor of these two antiviral strategies. The NS1 protein of YFV-DAK could also be more expressed and/or secreted than the one of YFV-17D.

Our NGS analysis detected more than 60 non-synonymous nucleotide differences along the genome of the two viral strains. These, together with the 8 nucleotide differences in the 3' untranslated region (UTR) between the 2 viral strains, could have functional consequences. The higher abundance of variants in YFV-17D genome as compared to YFV-DAK genome was unexpected since a recent study showed that the vaccine strains YFV-17D and YFV-FNV contained fewer variants than their respective parental strains [49]. However, our observations do not inform on the general variability of the genomes since they concern only a small number of nucleotides. Further analysis would be warranted to compare the genetic diversity of YFV-17D and YFV-DAK. Deep sequencing analysis of YFV-17D genome coupled to independent diversity measurements, such as the Simpson 1-D and Shannon entropy indexes, revealed that the vaccine strain lacks quasispecies diversity as compared to its parental strain Asibi [25]. This loss of genetic diversity has been proposed to contribute to YFV-17D attenuation in vaccinated patients [25]. Moreover, recent studies with Venezuelan equine encephalitis virus (VEEV), which belongs to the genus *Alphavirus*, have revealed that viruses able to disseminate in mosquitoes have an higher diversity than the ones that did not disseminate [50]. Thus, the poor genetic diversity of YFV-17D may contribute to its inability to infect and spread in *Ae*. *aegypti*.

Additional studies will be needed to identify the molecular mechanism(s) responsible for the low replication and dissemination of the YFV-17D vaccine strain in *Aedes* mosquito. These studies are essential to better understand the interactions between viruses and their vectors and can also contribute to the development of non-transmissible live-attenuated vaccines.

## Supporting information

S1 Fig YFV-17D, but not YFV-DAK, fails to overcome the midgut barriers of *Aedes aegypti* (second and third replicates). Mosquitoes were orally infected with 4x10^7^ PFU/mL of YFV-DAK (A and C) or YFV-17D (B and D). The relative amounts of organ-associated viral RNA were determined by RT-qPCR analysis and are expressed as genome equivalents (GE) per organ at 3, 5, 7, 10, 12 and 14 day post feeding (dpf). Total RNA was also extracted from several whole mosquitoes the same day of the feeding (black dots). Each data point represents the YFV titers of a single organ. The dashed lines indicate the limit of detection. Experiments were done three times independently. One representative experiment is shown in Fig 1 and the other two replicates are shown here.
(TIFF)

S1 Table Sequence read count and coverage for NSG samples. (TIFF)
