# Supplementary figures and images for: Midgut barriers prevent the replication and dissemination of the yellow fever vaccine in Aedes aegypti
Source: PLoS Negl Trop Dis. 2019 Aug 14;13(8):e0007299. doi: 10.1371/journal.pntd.0007299 (PMC6709925; doi:10.1371/journal.pntd.0007299)

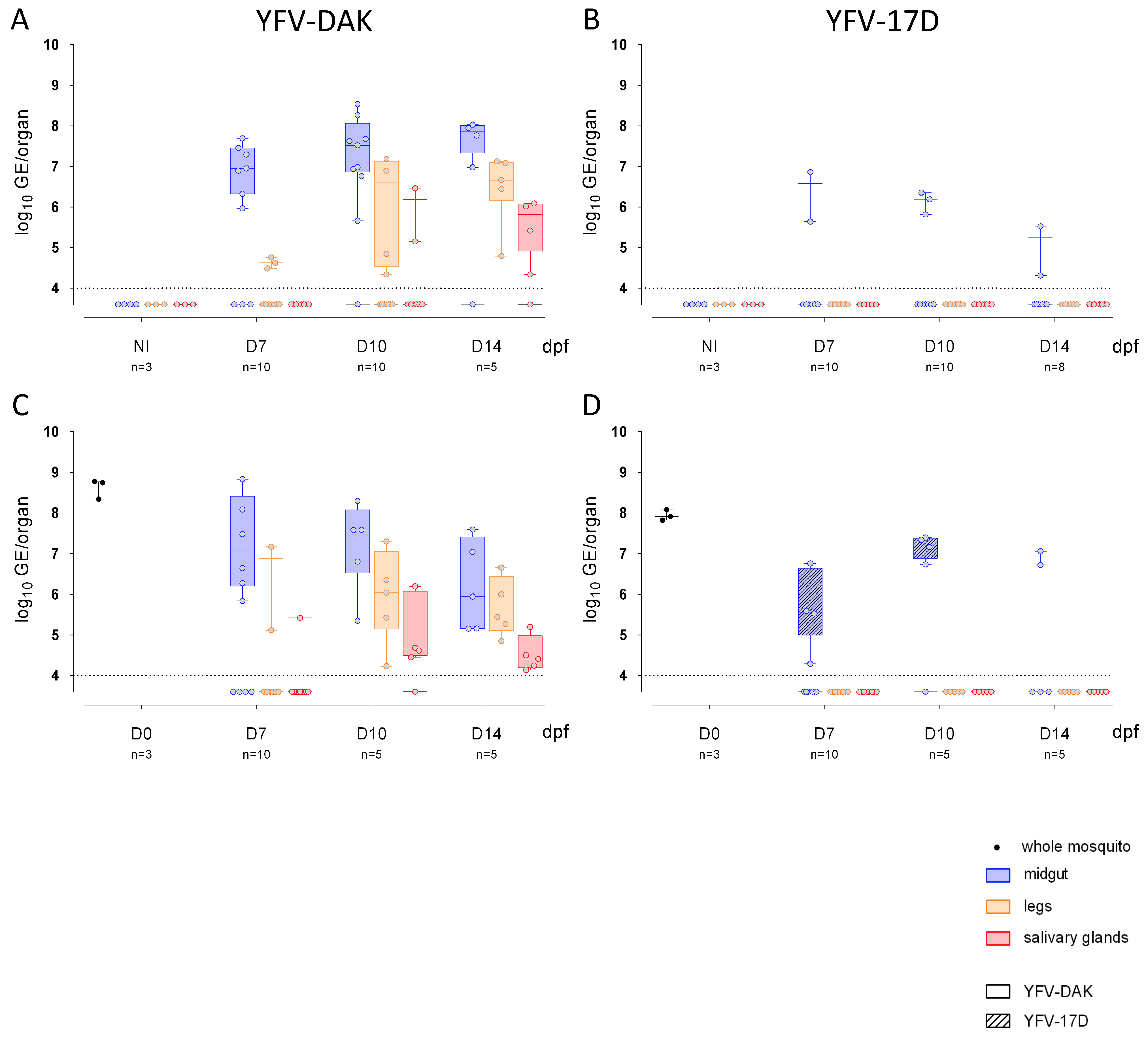

Supplement: S1 Fig — Mosquitoes were orally infected with 4x10^7^ PFU/mL of YFV-DAK (A and C) or YFV-17D (B and D). The relative amounts of organ-associated viral RNA were determined by RT-qPCR analysis and are expressed as genome equivalents (GE) per organ at 3, 5, 7, 10, 12 and 14 day post feeding (dpf). Total RNA was also extracted from several whole mosquitoes the same day of the feeding (black dots). Each data point represents the YFV titers of a single organ. The dashed lines indicate the limit of detection. Experiments were done three times independently. One representative experiment is shown in Fig 1 and the other two replicates are shown here. (TIFF) [file pntd.0007299.s001.tiff]

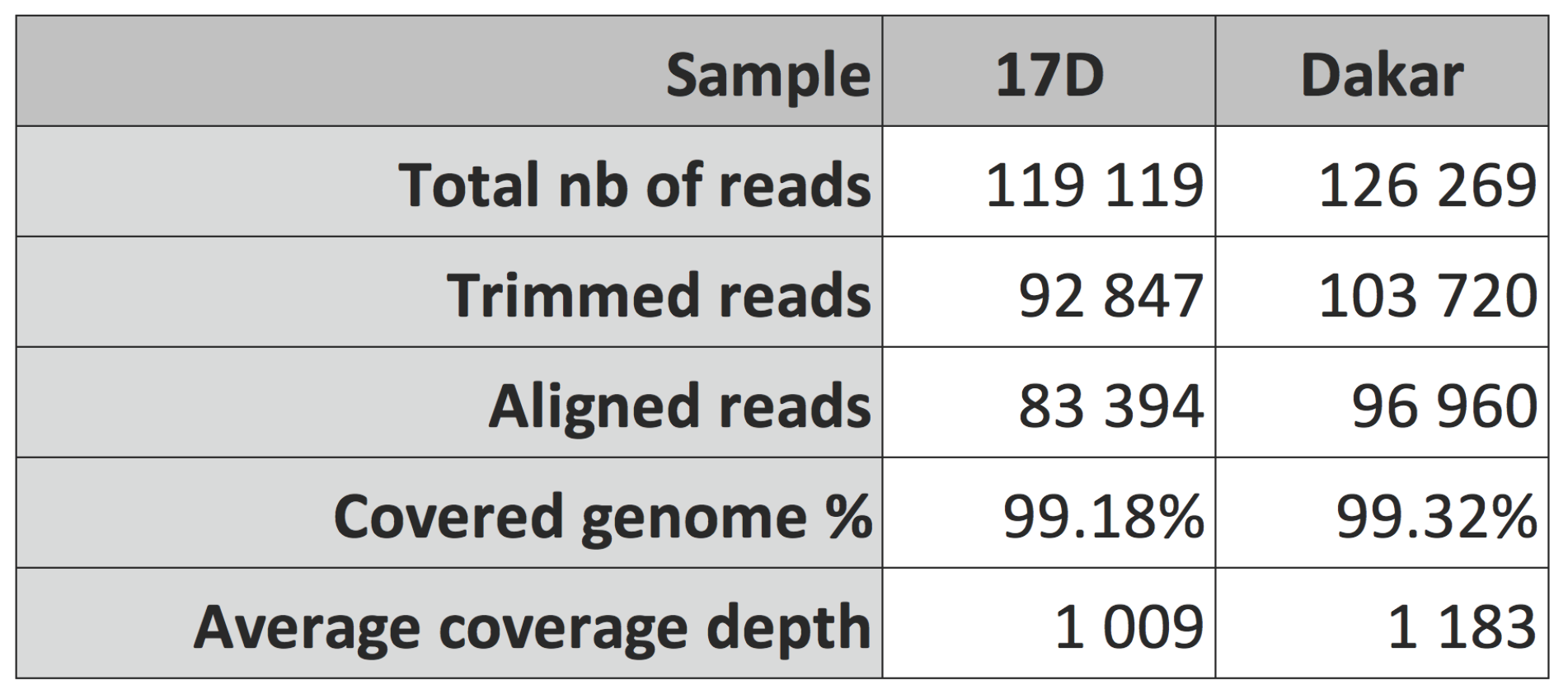

Supplement: S1 Table — (TIFF) [file pntd.0007299.s002.tiff]
